# Nanomedical approaches to deplete intracellular glutathione in oncology

**DOI:** 10.1039/d5sc09556e

**Published:** 2026-01-15

**Authors:** Javier Bonet-Aleta, Jose L. Hueso, Andrea Mosseri, Jesus Santamaria

**Affiliations:** a Yusuf Hamied Department of Chemistry, University of Cambridge CB2 1EW Cambridge UK jb2648@cam.ac.uk; b Instituto de Nanociencia y Materiales de Aragón (INMA), CSIC-Universidad de Zaragoza Zaragoza 50009 Spain jlhueso@unizar.es; c Networking Res. Center in Biomaterials, Bioengineering and Nanomedicine (CIBER-BBN), Instituto de Salud Carlos III 28029 Madrid Spain; d Department of Chemical and Environmental Engineering, University of Zaragoza 50018 Zaragoza Spain; e Instituto de Investigación Sanitaria (IIS) de Aragón 50009 Zaragoza Spain; f Escuela Politécnica Superior, Universidad de Zaragoza Crta. de Cuarte s/n 22071 Huesca Spain

## Abstract

Glutathione (GSH) plays a critical role in maintaining redox homeostasis and conferring chemoresistance to cancer cells, making it an attractive target for therapeutic intervention. Recent advances in nanomedicine have led to the development of diverse nanostructures capable of depleting GSH, either stoichiometrically or catalytically. These systems exploit the elevated GSH demand in tumors to selectively disrupt redox balance, enhancing reactive oxygen species (ROS) accumulation and improving the efficacy of conventional therapies. Approaches include metal-based nanocatalysts, GSH-responsive prodrugs, and stimuli-activated platforms using light or ultrasound for spatiotemporal control. Despite promising preclinical outcomes, key challenges remain, including limited mechanistic understanding, variability in GSH sensitivity across cancer types, and a lack of standardized assays to evaluate GSH-depleting efficiency. Addressing these gaps will require cross-disciplinary efforts bridging materials science, chemical biology, and catalysis. Such integration is essential for translating GSH-targeting nanomedicines into effective clinical tools against drug-resistant malignancies. This perspective provides an overview of some of the most promising nanomaterials explored in the current state of the art and discusses the main strategies and challenges relevant to future clinical translation.

## Abstract 2

Glutathione (GSH) plays a critical role in maintaining redox homeostasis and conferring chemoresistance to cancer cells, making it an attractive target for therapeutic intervention.

## Graphical abstract

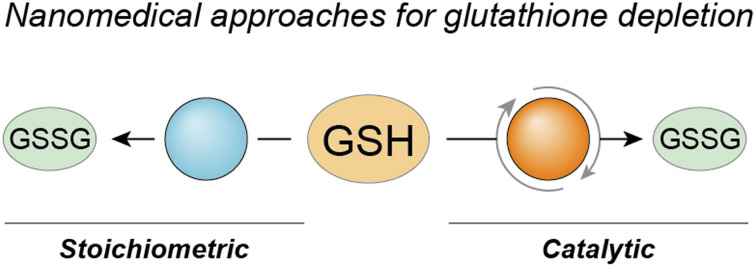

## 1 Introduction

Tumor growth leads to the formation of a region with distinct characteristics compared to the rest of the organism, namely the tumor microenvironment (TME). The TME exhibits a unique chemical profile that contributes to cancer progression and/or the development of resistance to certain therapies. Glutathione (GSH) represents one of the most remarkable examples of biomolecules that are significantly overexpressed across multiple types of cancer cells.^1,2^ The cytosolic concentration of GSH is typically in the millimolar (mM) range, whereas its extracellular levels rarely exceed the micromolar (µM) range.^3^ This pronounced gradient, along with the overexpression of GSH in malignant tissues, has inspired the development of switchable (on/off) systems that respond specifically to GSH-rich environments. Of particular interest are nanomaterials for GSH targeting, given their distinct advantages over conventional drugs based on small molecules. While traditional drugs generally act in a stoichiometric manner, nanostructures are designed to either deplete GSH levels or use GSH as a trigger to initiate therapeutic action, or both. The aim of this review is to provide a general overview on emerging nanomedicines that can exploit the large differences in GSH concentration between healthy and cancer cells to achieve a potent therapeutic action with less aggressiveness towards non-cancerous tissues.

## 2 Biological functions of GSH relevant to nanomedicine for cancer therapy

Beyond a mere difference in concentration, substantial evidence supports the critical dependence of cancer cells on GSH and the therapeutic potential of its depletion.^4–6^ GSH is a tripeptide combination of glutamate, cysteine, and glycine, with a range of biological functions primarily attributed to the reactivity of its thiol group.^7^

**Fig. 1:**
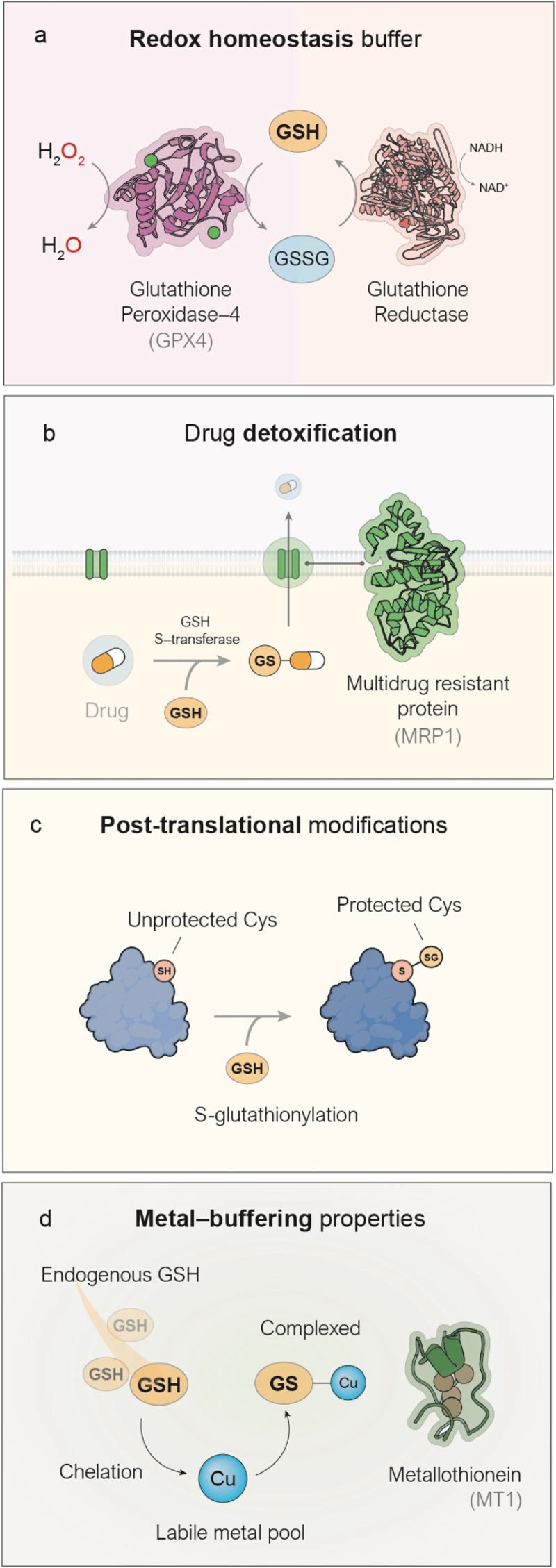
Key biological roles of GSH relevant to cancer therapy. (a) GSH is the main antioxidant in mammalian cells, serving as a cofactor for the enzyme GPX4, which converts harmful hydrogen peroxide (H_2_O_2_) into harmless water (H_2_O).^8^ (b) GSH possesses a highly reactive –SH group that interacts with various drugs to form conjugates (GSH–drug), which can then be processed and expelled from the cell by MRP1 proteins located in the cell membrane.^16^ (c) GSH protects exposed cysteine residues on proteins through a process called *S*-glutathionylation, which helps stabilize a variety of proteins.^17,18^ (d) The thiol group in GSH has a high affinity for transition metals such as zinc and copper, enabling the cell to use GSH to maintain intracellular metal homeostasis.^19,20^

Its most recognized role is as an antioxidant,^8^ mediating the elimination of ROS by acting as a substrate for the glutathione peroxidase (GPx) enzyme family, in a process that converts GSH and endogenous hydrogen peroxide (H_2_O_2_)—a byproduct of various biological processes—into oxidized glutathione (GSSG) and H_2_O (Fig. 1a). This mechanism is of special relevance in cancer metabolism due to the high oxidative stress characteristic of the TME.^9^ In contrast to cancer, GSH dysregulation in neurodegenerative diseases^10^ or liver^11^ diseases commonly occurs through downregulation. Factors such as the elevated metabolic activity which sustains cancer growth and development,^12^ activation of oncogenes such as RAS^13^ or MYC^14^ and the hypoxic conditions normally found in tumors,^15^ among others, are responsible for this oxidative stress which leads to upregulated GSH to maintain redox homeostasis in cancer cells. The nanomedicines discussed in the following sections of this review are designed to disrupt redox homeostasis by targeting GSH. Beyond its antioxidant function, GSH also fulfils additional roles of therapeutic relevance. GSH participates in the detoxification of xenobiotics, including several chemotherapeutic drugs,^21^ by reacting with them through the action of glutathione S-transferases to form GSH-drug conjugates.^22^ In a final step, these conjugates are actively transported out of the cell into the extracellular space by multidrug resistance proteins (MRP1),^16^ a process that consumes ATP (Fig. 1b) and indeed contributes to the development of chemoresistance in a variety of cancers. The reactivity of the thiol group in GSH can be exploited by the cell to accomplish post-translational modification of proteins, in a process known as glutathionylation^23^ (Fig. 1c). In this process, exposed cysteine residues on proteins can spontaneously form disulfide bonds with GSH, thereby blocking their reactivity in other biological pathways. This modification has been shown to alter the activity of key proteins involved in cancer cell survival, such as NF-κB^17^ and p53,^18^ among others. Lastly, another remarkable function of GSH is related to its large metal binding affinity especially for transition metals such as copper and zinc (Fig. 1d). Numerous pieces of evidence show that GSH acts as an intracellular metal buffer, with roles such as transferring metals to certain proteins, including metallothioneins.^19,20^ Thus, GSH plays a variety of survival-key roles in cancer cells which explains the high GSH levels generally found, a key factor to consider when designing metal-based nanoformulations, aimed at depleting GSH in cancer cells.

Typically, the difference in GSH concentration between healthy and cancerous cells is exploited by nanomedicines to enhance therapeutic selectivity. In particular, strategies that are activated above a certain GSH threshold are especially effective. Once GSH depletion is triggered, the process is especially harmful to tumours compared to healthy tissues since as we have shown, GSH is a key molecule involved in numerous cellular processes, particularly the detoxification of ROS, drugs, and metals. Thus, the design of nanomedicines primarily exploits the elevated GSH levels in cancer cells to unleash a therapeutic action that relies precisely on depletion of GSH.

The present review aims at providing a broad overview of current nanomedicines that target GSH for cancer therapy, based on the activity principles outlined (*vide supra*). We have classified them according to their reactivity patterns toward GSH, *i.e.*, whether they deplete GSH levels in a stoichiometric (Section 3) or catalytic manner (Section 4) (see Table 1). This distinction informs the design of nanomedicines, which in turn affects their therapeutic mechanisms and their potential application in the clinic. Rather than providing a comprehensive list of all GSH-depleting nanomedicines in the realm of cancer therapy, we aim to summarize the main applications of this strategy in chemical biology, catalysis and therapy, providing a vision of current developments and future perspectives in this field.

**Table 1:** Summary of GSH-depleting approaches comprising nanostructures

Modality	Type of nanoparticle		Pros	Cons
Stoichiometric (Section 3)	MnO_2_-based nanoparticles		Generation of Mn^2+^ ions allows for CDT, MRI or immunotherapy	Release of toxic Mn^2+^ in healthy tissue
	Organosilica nanoparticles		Biodegradable. Resistant to enzymatic degradation/acidic pH	Long term accumulation of Si in off-target tissue. Kinetics have to be characterized
	Polymers containing S–S bonds		Biodegradable. Encapsulation of drugs with different polarity	Long term biocompatibility needs to be studied
	Self-assembled nanoparticles containing S–S bonds		Improved biocompatibility. Biodegradable	Resistance against enzymatic degradation is a concern
	Pt(iv)-containing nanoparticles		Extensive knowledge of cisplatin in patients	Premature reduction in plasma. Kinetics regarding reduction need to be characterized
Catalytic (Section 4)	Oxidation	Copper containing nanostructures	Potent redox homeostasis dysregulation. Good selectivity in cancer cells	Premature degradation in plasma. Off-target toxicity
		Noble metals	Potent activity. Potential interaction with light	Significant systemic toxicity
		Photo- or sono-catalysts	Localized activity	Typically materials with poor biodegradation and potential systemic toxicity. Potential off-target activity due to local heating
	Transamination	Cu^2+^ containing nanostructures	Independent of O_2_ levels	Low kinetics. Premature degradation in plasma. Off-target toxicity

## 3 Nanomedicines for stoichiometric depletion of GSH

**Fig. 2:**
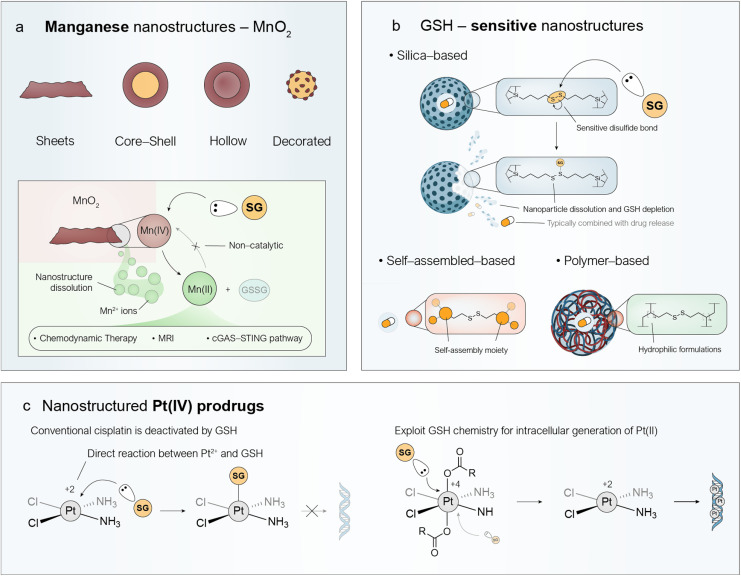
Nanostructured approaches for the stoichiometric depletion of intracellular GSH. (a) MnO_2_ can be engineered into a variety of nanostructures, including nanosheets, core–shell particles, hollow spheres, and decorated nanoparticles^25,26^ aimed at depleting intracellular GSH levels. (b) Schematic illustration of the dissolution process of Mn-NS through direct reaction with GSH, resulting in the release of Mn^2+^ ions. These ions are therapeutically relevant in several contexts, including chemodynamic therapy,^27^ MRI imaging,^28^ and activation of the cGAS-STING^29^ pathway for immunotherapy. (c) Different types of engineered nanoparticles—such as silica-based,^30^ self-assembled,^31–33^ or polymer-based systems^34^—incorporating cleavable disulfide bonds within their structures can yield GSH-responsive nanomedicines. Typically, therapeutic payloads are encapsulated within these nanoparticles to achieve a synergistic effect by combining GSH depletion through direct reaction with the disulfide bonds, and the payload release following cleavage of disulfide bonds that occurs preferentially within the TME driven by the high GSH levels. Mechanism of deactivation of conventional Pt(ii) drugs by GSH. Nanostructured Pt(iv) prodrugs generate active Pt(ii) species through a direct reaction with GSH (Step 1), thereby depleting intracellular GSH levels. GSH depletion helps prevent the deactivation of the active Pt(ii) species, allowing them to induce DNA damage effectively (Step 2).^35,36^

The thiol group in the cysteine residue of GSH exhibits high reactivity toward a broad range of electrophiles—a feature that nanomedicine can exploit in cancer therapy, either by depleting intracellular GSH concentrations or by triggering the release or activation of a drug encapsulated within a nanostructure. In this section, we discuss three main families of nanostructures: manganese dioxide nanostructures (Mn-NS) (Fig. 2a), GSH-sensitive nanostructures—including those based on silica, self-assembly, or polymers (Fig. 2b)—and nanostructured Pt(iv) prodrugs (Fig. 2c). Although all these systems operate *via* the same underlying mechanism (*i.e.* direct reaction with GSH), they exploit this reactivity for different therapeutic purposes.

### 3.1 Manganese dioxide nanostructures and their multiple roles as anticancer agents

The reactivity between Mn-NS and GSH for biomedical purposes was first explored by Deng *et al.* in 2011 to develop GSH sensors.^24^ The surface of luminescent upconversion nanoparticles was coated *in situ* with MnO_2_ nanosheets, which quenched their luminescence through an energy transfer process. Upon exposure to the GSH-enriched intracellular environment, the MnO_2_ on the surface was reduced to Mn^2+^, restoring the initial luminescence of the upconversion nanoparticle core and enabling indirect quantification of GSH. Since then, a wide variety of nanostructures based on Mn(iv) have been explored including nanosheets, core–shell, hollow or dots (Fig. 2a).^25,26^ Indeed, this versatility also enables the combination with other metals within the same platform to achieve synergistic effects. In terms of reactivity, Mn(iv) exhibits a high standard reduction potential (*E*^0^_Mn(iv)/Mn(ii)_ = +1.23 V) and readily reacts with two molecules of GSH to form GSSG and Mn(ii). However, the Mn(ii) species do not remain confined within the nanostructure; instead, Mn-NS generally dissolve intracellularly, releasing Mn^2+^ ions (Fig. 2a). It is important to note that these Mn^2+^ ions are not directly reoxidized to Mn^4+^ owing to the high reduction potential *E*^0^ required, and the oxidation of GSH proceeds stoichiometrically rather than catalytically. Nevertheless, this is not necessarily a disadvantage, as the release of Mn^2+^ ions within cancer cells can play several promising roles: (i) promote Fenton-like reactions, (ii) enable magnetic resonance imaging (MRI) and (iii) activate the stimulator of interferon genes (cGAS-STING) pathway for immunotherapy (Fig. 2a).^27–29^

**Fig. 3:**
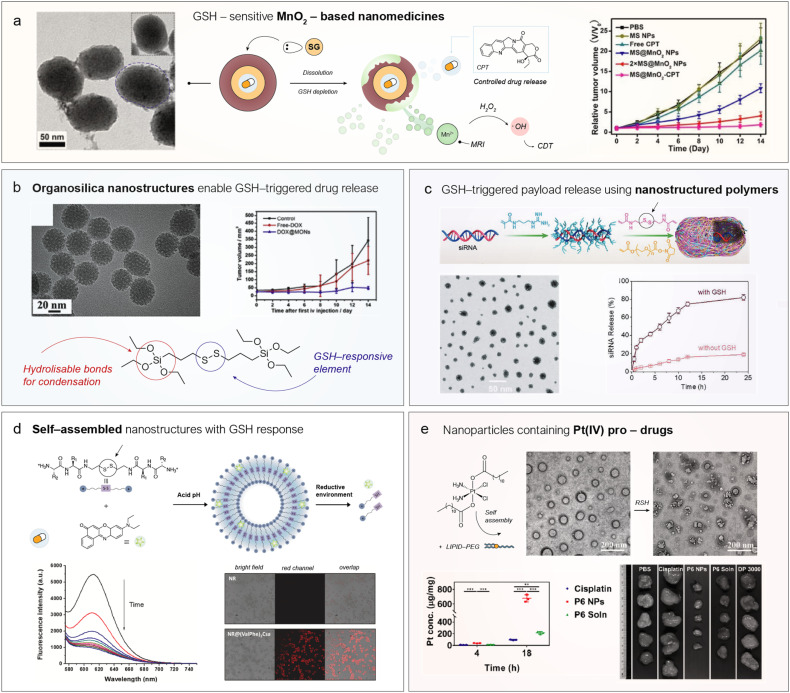
Representative examples of nanomedicines that exploit GSH reactivity for cancer therapy. (a) MnO_2_-coated mesoporous silica nanoparticles encapsulating camptothecin (CPT) can simultaneously deplete GSH *via* direct reaction, release Mn^2+^ to disrupt redox homeostasis through Fenton-like catalysis, and enable GSH-dependent drug release. Left: Transmission electron microscopy (TEM) images of MnO_2_-containing nanomedicines. Right: Tumor size monitoring in nude mice bearing U87MG tumors. Reproduced from ref. 38 with permission from Wiley-VCH Verlag GmbH & Co. KGaA, Weinheim, copyright 2018. (b) Mesoporous organosilica nanoparticles with drug-release capability triggered by GSH through direct cleavage of disulfide (S–S) bonds. Left: Representative TEM images of the organosilica nanoparticles. Right: Tumor size evolution in nude mice bearing 4T1 tumors. Reproduced from ref. 53 with permission from Elsevier, copyright 2018. (c) Scheme of the fabrication of a polymeric nanomedicine containing cleavable disulfide bonds to protect and release a therapeutic siRNA. Left: TEM images of the polymerized nanocapsules. Right: GSH-dependent release of siRNA. Reproduced from ref. 54 with permission from Wiley-VCH Verlag GmbH & Co. KGaA, Weinheim, copyright 2020. (d) Nanovesicles formed through the self-assembly of peptides containing a cysteamine unit for releasing Nile Red in the presence of large levels of GSH. Left: Fluorescence spectra of Nile Red at different times upon incubation with GSH. Right: Fluorescence microscopy images showing the internalization of Nile Red in cancer cells. Reproduced from ref. 33 with permission from American Chemical Society, copyright 2023 and from ref. 32 with permission from Elsevier, copyright 2023. (e) Incorporation of Pt(iv) centers into nanoparticles through a self-assembly process assisted by the axial ligands of the coordination complex. TEM images of the Pt(iv)-containing nanoparticles before and after the reduction of Pt(iv) to Pt(ii). Left: Internalization kinetics of different Pt-containing drugs reveal preferential accumulation in Pt when administrated in nanoparticles. Right: Tumor size at sacrifice under different treatments showed a clear reduction with Pt(iv) NP treatment. Reproduced from ref. 55 with permission from American Chemical Society, copyright 2019.

First, Mn^2+^ ions can catalyze the decomposition of endogenous H_2_O_2_ into highly toxic ˙OH radicals, ultimately inducing cell death.^37^ This reaction (a Fenton-like reaction) represents the foundations of chemodynamic therapy (CDT),^27^ an emerging therapeutic modality that involves using Fenton or Fenton-like reactions, typically triggered by nanostructured catalysts, to produce ROS directly inside tumor cells. Li *et al.*^38^ were pioneers in leveraging the dissolution of MnO_2_ by GSH to produce Mn^2+^*in situ* and disrupt the redox homeostasis in tumors formed by U87MG cells. They designed a nanomedicine consisting of a mesoporous silica nanoparticle—with a large surface area to adsorb and encapsulate camptothecin (CPT)—then coated it with a MnO_2_ shell (Fig. 3a, *vide infra*). Upon degradation of the MnO_2_ by intratumoral GSH, CPT is released; thus, the direct reaction between GSH and MnO_2_ not only depletes GSH levels but also triggers the release of a drug. In addition, the released Mn^2+^ ions can generate highly toxic ˙OH radicals, continuing to disrupt the redox balance of the cancer cells. Importantly, the authors identified the presence of HCO_3_^−^ ions as a key factor facilitating catalysis, likely by chelating Mn^2+^ ions and modifying the *E*^0^, thereby enabling easier regeneration of the active species^25^ (Fig. 3a). The combination of enhanced oxidative stress and CPT treatment led to a synergistic tumour size reduction (Fig. 3a). Since then, multiple combinations have been developed to exploit the Fenton-like chemistry of Mn^2+^ ions released from Mn-NS. For example, Fu *et al.*^39^ combined the enzyme glucose oxidase (GOx) with nanostructured MnO_2_ within the same platform. GOx catalyzed the oxidation of endogenous glucose into gluconic acid and H_2_O_2_, which could subsequently be transformed into ˙OH radicals by Mn^2+^ ions generated through the GSH-assisted dissolution of MnO_2_. In this way, GOx acted synergistically with MnO_2_ by increasing endogenous H_2_O_2_ levels, providing additional fuel for the Fenton-like reaction catalyzed by Mn^2+^. Other enzymes which belong to the oxidase family, such as lactate oxidase,^40^ have also been explored with the same purpose.

Secondly, Mn^2+^ ions have five unpaired electrons, making them paramagnetic and influencing the longitudinal relaxation time (*T*_1_) of nearby H_2_O molecules, which can be exploited for MRI imaging. After Mn-NS are internalized into tumors and dissolved through a direct reaction with GSH, the released Mn^2+^ ions can enhance the MRI signal. To increase the selectivity of imaging, Mn^2+^ releasing structures have been combined with tumor-targeting agents such as small molecules (*e.g.*, folic acid,^41,42^ hyaluronic acid^28^) or coated with cell membranes^43,44^ to evade the immune system and facilitate tumor accumulation.

Finally, Mn^2+^ ions are biologically active and capable of interacting with various cellular pathways. Among these, stimulation of the cGAS-STING pathway is particularly attractive for therapeutic purposes, as it ultimately triggers a strong immune response that enhances the body's ability to recognize and attack cancer cells.^45^ Specifically, the enzyme cyclic GMP–AMP synthase (cGAS) senses DNA leakage into the cytoplasm—a hallmark of cancer cells—and produces a signaling molecule called cGAMP. Mn^2+^ ions enhance the sensitivity and activity of cGAS in this process.^29^ The resulting accumulation of cGAMP then activates the STING protein, initiating an immune response.^46^ The ability of Mn^2+^ ions to activate cGAS was discovered in 2018 by Zhengfan Jiang's laboratory,^29^ and since then, multiple nanoformulations containing MnO_2_ or Mn-containing metal–organic frameworks (MOFs)^47,48^ have been shown to induce strong immune responses in cancer therapy. A comprehensive overview of the Mn-based nanostructures that activate the cGAS-STING pathway was published in 2023 by Zhang *et al.*^49^ The production of Mn^2+^ ions following reaction with GSH may also lead to undesired off-target toxicity. Particular attention should be paid to Mn-containing nanomedicines intended for brain cancer therapy, given the well-documented neurotoxicity of Mn.^50^ Excess Mn from nanoparticles can promote several harmful processes, including neuroinflammation, mitochondrial oxidative damage through Mn accumulation, and dopamine oxidation, among others.^51^ However, most of these neurotoxic effects arise from long-term systemic exposure to Mn^52^—typically over months or years—and may be less pronounced during short-term clinical treatments with Mn-based nanomedicines. We therefore encourage researchers working with these GSH-depleting nanomaterials to carefully consider this potential source of toxicity, especially in preclinical mouse studies, to share any observable hallmarks of neurotoxicity with the community, and to include postmortem histological examination of the brain—such as hematoxylin and eosin (H&E) staining.

In summary, the direct reaction of Mn-NS with GSH not only depletes this crucial biomolecule in the TME, but also enables the *in situ* production of Mn^2+^ ions. These ions contribute to the therapeutic efficacy of Mn-NS by generating hydroxyl radicals (˙OH), stimulating the cGAS-STING pathway, and serving as MRI contrast agents. Given the dual role of Mn-NS in both depleting the GSH pool and generating Mn^2+^ ions, further progress in this technology will require a deeper understanding of the pathways cancer cells use to regulate excess Mn^2+^. Such insights could be leveraged to design strategies that integrate Mn-NS and bioactive molecules able to inhibit the cellular detoxification pathways for Mn^2+^. These agents would be released upon GSH-mediated dissolution of Mn-NS, thereby prolonging the intracellular half-life of Mn^2+^ and enhancing its therapeutic impact.

### 3.2 GSH-sensitive nanostructures to enable simultaneous GSH depletion and drug release

Given the reactivity of the thiol group in the cysteine residue of GSH, it is possible to design responsive nanostructures that break down and degrade in response to high GSH concentrations, characteristic of the TME. This dual responsiveness allows controlled payload release while concurrently lowering intracellular GSH levels, creating attractive synergies for cancer therapy. In most cases these nanostructures contain disulfide bonds (S–S), which can be cleaved through direct reaction with GSH. Other linkages, such as diselenides (Se–Se),^56,57^ are also employed due to their lower bond dissociation energy (Se–Se: 172 kJ mol^−1^) compared to disulfides (S–S: 240 kJ mol^−1^),^58^ making them similarly reactive toward GSH; however, they are less widely explored. Fig. 2c displays the three main types of nanoparticles according to the structural elements that incorporate the disulfide (S–S) GSH-responsive bonds: organosilica, self-assembled, and polymeric nanoparticles.

Organosilica nanoparticles are synthesized using a building block analogous to tetraethyl orthosilicate (TEOS), commonly used in the Stöber method, but incorporating a disulfide bond (Fig. 3b).^59,60^ Under basic or acidic conditions, the –OCH_2_CH_3_ groups can be hydrolysed, allowing the silica to condense through Si–O–Si bonds. However, when disulfide-containing molecules such as bis-[3-(triethoxysilyl)-propyl]-disulfide (BTSPD) are added to the synthesis medium, the corresponding organic moieties can be intercalated, allowing the particles to disassemble. Porosity can be tuned by adding surfactants, yielding mesoporous nanoparticles suitable for encapsulating and delivering therapeutic payloads. These payloads can be released upon direct reaction of GSH with the disulfide bonds.^53,61^

The pore diameter plays a crucial role in GSH reactivity. At high intracellular GSH concentrations, large pores facilitate fast diffusion of GSH into the nanoparticles and toward the S–S bonds, resulting in quicker bond cleavage and particle degradation.^62^ Yu *et al.* synthesized several mesoporous organosilica nanostructures, including spheres and rods.^53^ The nanoparticle pore size was tuned by adjusting the ratio of silica (TEOS) to organosilica (bis[3-(triethoxysilyl)propyl]disulfide (BTSPD)) precursors. Doxorubicin (DOX) was loaded into the mesopores, and upon exposure to 10 mM GSH, approximately 70% of the DOX was released within 48 hours. In contrast, inorganic mesoporous silica nanoparticles showed minimal response to GSH, which highlights the known limitations in their biodegradability. This nanomedicine was capable of blocking tumor growth (Fig. 3b), whereas an equivalent dose of free DOX had only a moderate effect, underscoring the importance of TME-enhanced drug delivery for effective therapy.

A major advantage of these nanomedicines lies in their potential for combination cancer therapy. Significant therapeutic synergies can be achieved when GSH depletion is coupled with the release of a payload that exploits the disruption of redox homeostasis. For instance, Ma *et al.*^63^ loaded mesoporous organosilica nanoparticles with metals such as Cu or Zn, which generated highly toxic ˙OH species *via* Fenton-like reactions, as well as cisplatin—a drug that can be deactivated by GSH, as discussed in the next section. The simultaneous GSH depletion, ROS production, and cisplatin delivery resulted in potent cytotoxicity against A549 cancer cells. Other cytotoxic payloads include enzymes such as GOx,^64^ nucleic acids,^65^ antibodies^66^ or CRISPR genome editors (Cas9/sgRNA).^65^ Future directions in this area are expected to focus on the design of organosilica precursors with disulfide bonds engineered to react more selectively with GSH, minimizing premature payload release in healthy tissues. The rich chemistry of organosilica allows for a variety of possibilities in this respect (see *e.g.* the incorporation of nucleic acids into organosilica nanoparticles^67^).

The introduction of a single central pore, yielding a hollow morphology, has been reported to reinforce the organosilica framework.^68^ De Cola's and Santamaria's groups recently addressed this challenge by tuning the organosilica matrix to exhibit GSH concentration-dependent sensitivity.^60^ Specifically, they found out that replacing the conventional TEOS precursor with a more compact tetramethyl-orthosilicate (TMOS) produced nanoparticles resistant to low concentrations of reducing agents, while enabling payload release preferentially at the elevated GSH levels characteristic of the TME. Moreover, both the absolute amounts of silica and organosilica precursors, as well as their relative ratio, were found to critically influence redox stability.^60^ Future efforts should explore how varying combinations of silica and organosilica precursors—ranging from those that produce less GSH-selective frameworks to those that generate more robust structures—can be exploited to design nanoparticles that are both selective for the TME and capable of efficient *in situ* payload release. In terms of additional safety considerations, off-target internalization and degradation of silica or organosilica nanoparticles also deserve study, as these processes may lead to long-term accumulation of silicon-containing species in healthy tissues (as an example, accumulation for >12 days in cell culture has been reported^69^). Such accumulation can trigger undesired cellular effects, including lysosomal dysfunction.^70^ Beyond cellular retention, we found a study led by Henderson *et al.*^71^ particularly relevant when addressing the immunogenicity of organosilica nanoparticles, as it reports a size- and time-dependent interaction of organosilica nanoparticles with immune cells. This aspect should be carefully evaluated depending on the intended application and therapeutic strategy. Finally, as of 2023, eleven clinical trials had already assessed the safety profile of orally administered silica nanoparticles, finding good tolerability with no serious adverse effects,^72^ providing a promising foundation for the further clinical translation of organosilica-based nanomedicines.

GSH-responsive polymeric nanoparticles provide greater flexibility in synthesis, although they generally lack the structural features of organosilica nanoparticles, particularly in terms of porosity. The building blocks typically consist of tri-functional molecules containing two reactive groups to allow polymerization—such as acrylamides—at each end, and a GSH-responsive group (usually a disulfide bond) positioned within the structure.^34,73,74^ These nanoparticles are commonly prepared *via* nanoprecipitation^75^ and typically carry drugs that are either unstable in plasma (siRNA, organometallic complexes^76,77^) or hydrophobic. Zou *et al.*^54^ developed GSH-responsive nanocapsules containing siRNA with a diameter of 25 nm (Fig. 3c). They polymerized an acrylamide-containing siRNA with a disulfide-containing molecule to yield nanocapsules that protected the siRNA from degradation in the presence of FBS and enabled its release under 10 mM GSH conditions (Fig. 3c). This protection increased the siRNA's elimination half-life in plasma from 5 minutes (for naked siRNA) to 46 minutes (for encapsulated siRNA). The organic nature of the components used in these nanomedicines provides chemical versatility, enabling the introduction of new functionalities. For instance, Zhan *et al.*^78^ created a GSH-responsive nanomedicine by appending a disulfide bond to a fluoropolymer, enhancing the encapsulation of hydrophobic drugs. This system enabled the co-encapsulation of hydrophobic DOX and the p53 gene, facilitating combined chemo- and gene therapy upon GSH exposure.

Another strategy involved covalently attaching the active drug to the polymer, rather than relying solely on encapsulation, using a GSH-cleavable bond. Yin *et al.*^79^ employed this approach to design a polymeric nanomedicine by linking CPT to a disulfide-containing monomer and synthesizing the final polymer *via* reversible addition-fragmentation chain-transfer (RAFT) polymerization. This method minimized passive desorption that would cause premature CPT release in healthy tissue, thereby enhancing the safety profile of the nanomedicine. Ye *et al.*^80^ reported a related system where 10-hydroxycamptothecin (HCPT) was covalently conjugated to a disulfide cross-linker, enabling precise and controllable release. This approach not only enhanced the tumour bioaccumulation of HCPT, but also prolonged its circulation half-life.

Self-assembly chemistry can also be used to construct nanoparticles, as an alternative to polymerization (Fig. 2c). One of the main advantages of this strategy is the excellent biodegradability of such nanomedicines,^81,82^ as the forces required to disassemble them are significantly weaker than the covalent bonds found in organosilica or polymer-based systems, while preserving GSH-responsiveness. An interesting class of self-assembled GSH-responsive compounds is based on peptides,^31,33^ whose disassembly results in biocompatible molecules. Bernal *et al.*^32^ developed a peptide derivative capable of self-assembling into nanovesicles *via* intermolecular hydrogen bonding and π–π stacking (Fig. 3d). A hydrophobic photodrug, Nile Red, was encapsulated within the vesicles during the self-assembly process at acid pH, and was released upon exposure to GSH (Fig. 3d). This enabled the internalization of the drug into cancer cells, whereas the free drug showed negligible uptake (Fig. 3d).

Promising future directions in GSH-responsive nanomedicines include the development of improved building blocks containing disulfide bonds—whether organosilica, polymeric, or self-assembled—with finely tuned properties that confer selective activation under the elevated GSH levels, characteristic of many cancers. This approach would also mitigate the premature release of toxic payloads in healthy tissues, leading to more efficient and safer therapeutic outcomes.

### 3.3 Intracellular activation of nanostructured Pt(iv) prodrugs by direct reaction with GSH

The nucleophilic thiol group of GSH plays a key role in the deactivation of Pt-based chemotherapeutics within the TME, contributing to chemoresistance in multiple cancer cell lines.^83^ The mechanism of action of cisplatin and its derivatives involves preferential binding to the guanine bases of DNA,^84^ a process that requires platinum in the +2 oxidation state. This interaction typically activates p53 and triggers apoptosis, leading to cancer cell death. However, GSH, at the high intracellular concentration of the TME, tends to react with Pt(ii), forming reduced and inactive GS–Pt conjugates,^85^ which can be detoxified through MRP1, a membrane-associated efflux transporter (Fig. 1b).^4^

Pt(iv) prodrugs are designed to undergo direct reduction by GSH, thereby yielding the active Pt(ii) species in a single redox step (Fig. 2d). Another limitation of molecular cisplatin lies in its internalization pathway, which primarily occurs *via* High Affinity Copper Uptake Protein 1 (CTR1),^86^ a membrane transporter associated with a relatively slow uptake process. In contrast, nanoparticles can enter cancer cells exploiting the active transport and retention (ATR) principle,^87^ which suggests that NPs penetrate tumors through mechanisms involving active transport such as endocytosis^88^ or pinocytosis,^89^ typically resulting in faster intracelluar accumulation of drugs. To address these issues, a nanostructured cisplatin(iv) analogue was developed in 2019 by Ling *et al.*^55^ This compound possesses the dual ability to directly react with GSH, yielding the active cisplatin(ii) species, and to self-assemble into ∼100 nm nanoparticles in the presence of lipid-PEG (Fig. 3e).

Upon exposure to thiols, the carboxylate ligands coordinated to Pt(iv) are released *via* reduction to Pt(ii), leading to nanostructure disassembly and the liberation of active Pt(ii) species. The nanostructured Pt(iv) analogue exhibited a tenfold increase in cellular Pt uptake compared with molecular cisplatin (Fig. 3e). In mice bearing cisplatin-resistant A2780 tumors, treatment with molecular cisplatin resulted in minimal tumor shrinkage, whereas administration of the nanostructured Pt(iv) led to a significant reduction in tumor volume. These findings highlight the therapeutic potential of nanostructured Pt(iv) in enhancing drug internalization and overcoming GSH-mediated deactivation (Fig. 3e). Improved formulations have included covalent attachment of perfluorocarbon chains to the Pt(iv) centre, enhancing plasma membrane interactions and drug delivery efficiency.^90^ Pt(iv) complexes have also been incorporated into different nanostructure formulations such as nanogels,^91^ polymers^92,93^ or DNA nanoarchitectures,^94^ or peptide-conjugated systems for tumor targeting^95,96^ and even red blood cell membrane-coated nanoparticles.^97^ These formulations consistently demonstrated the ability to overcome chemo-resistance mechanisms primarily associated with GSH. A comprehensive survey of the numerous nanostructured delivery systems used to encapsulate Pt(iv) is available in a recent review by Zhou *et al.*^36^

Future developments in this area are likely to focus on modifying the chemical environment of Pt(iv) to tune its reduction and activation pathways in such a way that selectivity toward GSH is enhanced, enabling activation specifically at tumor sites while minimizing reaction with other reducing agents of even with GSH present at lower concentrations in healthy cells. Additional therapeutic synergies such as immunotherapy and sonodynamic therapy could further amplify the efficacy of Pt(iv) prodrugs through a combination of GSH depletion and DNA damage. The main rationale for using Pt(iv) prodrugs is to enhance the stability of the metal center before it reaches the intended target. In patients, cisplatin interacts strongly with plasma proteins, particularly human serum albumin, which can lead not only to ligand exchange and protein binding but also to the formation of Pt-containing nanoparticles.^98^ Such processes can alter biodistribution, reduce the amount of active drug reaching the tumor, and contribute to systemic toxicity. Therefore, careful evaluation of the structural and redox stability of Pt(iv)-containing nanomaterials in biologically relevant media is essential. The use of ^195^Pt NMR^99^ in both plasma and serum under physiologically relevant conditions can yield valuable information regarding potential reduction, ligand exchange, or aggregation processes induced by proteins and biomolecules.

Overall, given its stoichiometric reactivity with cleavable moieties in nanostructures, GSH can be effectively exploited as a redox-active agent for the selective release of therapeutics in the TME. Based on the studies discussed in this perspective, current research efforts in nanomedicine are driving innovation toward improved selectivity for GSH, both in terms of molecular specificity and intratumoral concentration. Nevertheless, further investigation is still needed to determine (i) which oxidized forms of GSH are generated upon drug release, (ii) how these species are metabolized by the cell, (iii) what by-products are released from the nanomedicine after its action (for example, Mn^2+^ ions or silicon), and (iv) the potential side effects associated with these by-products.

## 4 Catalytic consumption of GSH to disrupt the redox homeostasis

One of the major limitations of conventional drugs is their poor accumulation within tumors.^100^ Numerous strategies have been explored to enhance tumor targeting through nanotechnology. However, a landmark 2016 meta-analysis by Wilhelm *et al.*,^101^ which examined over 200 preclinical studies, found that only about 0.7% (median) of the injected nanoparticle dose actually reached solid tumors. In fact, delivery efficiencies exceeding 5% are still considered exceptional and this remains a critical obstacle for the clinical translation of nanomedicine. Given these discouraging figures, there is growing interest in developing catalytic nano-agents able to sustain therapeutic effects over time. The key advantage of a catalyst over a conventional drug is that, even if only a small fraction of the administered catalyst reaches the tumor, it will perform its therapeutic action until the substrate is depleted or the catalyst is deactivated. In this way, much more than one therapeutic event occurs per catalytic unit, generating an extended action that may balance a lack of delivery efficiency. This approach could overcome the limitations of traditional drugs such as cisplatin, which acts by stoichiometrically reacting with DNA through a single event.

**Fig. 4:**
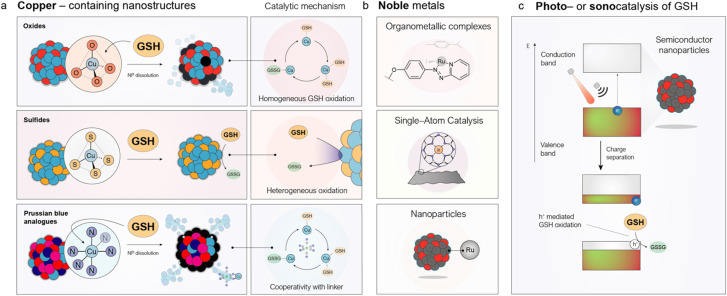
Nanostructured catalysts to cyclically deplete GSH levels in tumors. (a) Different copper-containing catalysts can oxidize GSH through various mechanisms depending on their composition. Oxides^108^ and PBAs^114^ undergo dissolution accelerated by GSH, with the catalytic cycle occurring in the aqueous phase, whereas copper sulfides catalyze GSH oxidation in a heterogeneous manner.^117^ (b) Noble metals capable of catalytically oxidizing GSH are incorporated into various nanomedicines, including organometallic complexes bearing phenazopyridine ligands,^118^ single-atom catalysts,^97^ and nanoparticles.^119^ (c) Schematic representation of the band structure of semiconductor nanoparticles. Electrons in the valence band can be excited to the conduction band directly by light or indirectly by ultrasound with appropriate energy, generating an electron–hole (e^−^/h^+^) pair. Subsequently, an electrophilic GSH molecule can consume the h^+^, leading to its oxidation to GSSG.

To this end, several nanostructured catalysts have been designed and developed to deplete GSH levels as a therapeutic mechanism in cancer. In this section, we identify three families of nanomedicines that catalytically deplete GSH in the TME, differing in composition and mode of action: copper nanostructures, noble-metal catalysts, and photo- or sonocatalysts (Fig. 4).

### 4.1 Copper nanostructures catalytically oxidize GSH through a structure-dependent mechanism

Copper is a pivotal transition metal in numerous redox-driven catalytic transformations, owing to its facile switch between +1 and +2 oxidation states. Among these processes, the ability of Cu(ii) ions to oxidize thiols has been recognized for decades. Although the precise mechanism of GSH oxidation by Cu(ii) is not fully elucidated yet, it is generally accepted that the reaction involves the initial formation of a Cu–GSH complex which is further oxidized into GSSG using dissolved O_2_. The first report employing a copper-containing nanostructure to deplete GSH in the TME was published only in 2016 by Ju *et al.*^102^ They decorated photoactive g-C_3_N_4_ with Cu(ii) centres, which could catalytically reduce GSH levels, thereby weakening the cancer cells' ability to neutralize ROS generated upon light irradiation of carbon nitride (g-C_3_N_4_) nanosheets. A key feature of this catalytic cycle is the formation of reduced Cu(i) species, which can act as Fenton-like catalysts by converting H_2_O_2_ into highly toxic ˙OH, thereby enabling *in situ* CDT.^103,104^ This efficiency arises from the markedly higher kinetic constant of the Fenton-like reactions for Cu(i) compared to Cu(ii), where *k*_Cu(i)_ ≫ *k*_Cu(ii)_.^105^ As a result, copper species can establish a catalytic synergy in environments enriched with both GSH and H_2_O_2_, such as the TME, enabling simultaneous GSH depletion and ˙OH generation that disrupts redox homeostasis in cancer cells.^106^ Further evidence indicates that dysregulation of GSH levels *via* copper oxidation is directly linked to a recently discovered form of cell death known as cuproptosis, which can be leveraged for therapy. Lu *et al.*^107^ developed nanoparticles that co-encapsulated Cu(ii) ions and celastrol, an inhibitor of nuclear factor kappa-B (NF-κB) involved in GSH biosynthesis. The simultaneous Cu-catalyzed depletion of GSH and inhibition of its synthesis induced cuproptosis in tumor cells, resulting in strong therapeutic activity in mice.

**Fig. 5:**
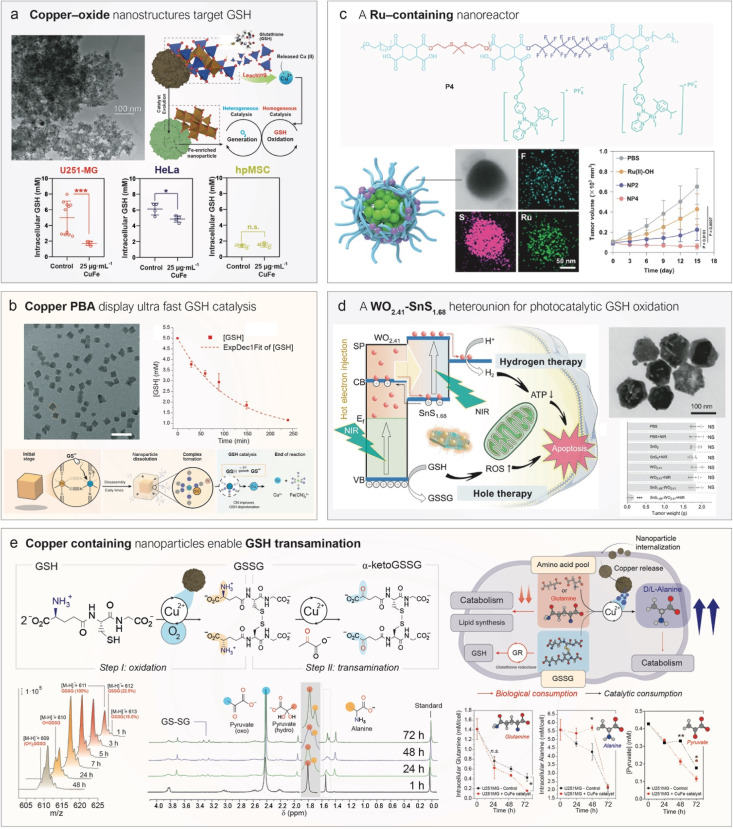
Representative examples of nanocatalytic medicines towards GSH oxidation. (a) A copper–iron oxide nanoparticle catalytically consumes GSH in cancer cells following copper leaching from the solid phase. Left: TEM image of CuFe_2_O_4_ nanoparticles. Right: Proposed working mechanism, in which the homogeneous catalysis by leached copper ions is coupled with heterogeneous catalytic O_2_ generation facilitated by the remaining iron-enriched nanoparticle. Reproduced from ref. 106 with permission from Elsevier, copyright 2022; and reproduced from ref. 108 with permission from Royal Society of Chemistry, copyright 2022. (b) A copper-containing PBA nanocube consumes GSH with an outstanding turnover frequency *via* a mechanism involving copper leaching and interaction with the nanocube's ligands. Left: TEM image of nanocubes. Right: GSH concentration over time in the presence of catalytic amounts of nanocubes. Reproduced from ref. 114 with permission from Wiley-VCH Verlag GmbH & Co. KGaA, Weinheim, copyright 2025. (c) A self-assembled polymeric nanoparticle bearing organometallic ruthenium complexes acts as a nanoreactor for catalytic GSH oxidation. Right-top: Representative TEM-EDS (energy-dispersive X-ray spectroscopy) mapping of the nanomedicine. Right-bottom: Antitumor effects of different nanoparticle formulations (NP2 and NP4) compared to the Ru(ii)–OH complex. Reproduced from ref. 118 with permission from Springer Nature, copyright 2024. (d) Schematic therapeutic mechanism of action of a WO_2.41_@SnS_1.68_ semiconducting nanoparticle which under 808 nm irradiation is able to oxidize GSH. Top right: TEM image of WO_2.41_@SnS_1.68_. Bottom right: tumor size under different treatment conditions. Reproduced from ref. 135 with permission from Springer Nature, copyright 2021. (e) Copper ions catalyze GSH/GSSG transamination using pyruvate as α-keto acid. Reaction pathway towards GSSG transamination. In the first step, Cu(ii) ions catalyze GSH aerobic oxidation, yielding GSSG as the product. Then, Cu(ii) ions are able to catalyze GSSG/pyruvate transamination into α-keto GSSG and alanine. Production of α-keto GSSG and alanine, respectively, against reaction time using CuFe_2_O_4_ nanoparticles as a catalyst. Reproduced from ref. 136 with permission from American Chemical Society, copyright 2024.

Our group has contributed to this field by developing copper-containing nanocatalysts for GSH oxidation and elucidating the mechanisms underlying their activity (Fig. 4a). For instance, Bonet-Aleta *et al.* demonstrated that Cu oxide-based nanoparticles dissolve in the presence of high GSH concentrations, releasing most of the Cu content of the nanoparticle that then acted as the true catalytically active species in solution (Fig. 5a).^108^ Characterization *via* mass spectrometry and ^1^H NMR of controlled mixtures of CuCl_2_, as a source of Cu^2+^ ions, and GSH demonstrated the formation of Cu-glutathione complexes which were further oxidized to GSSG. This finding revealed that GSH oxidation proceeded *via* a homogeneous, rather than a heterogeneous, process—an insight with important implications for both delivery and biological activity. Interestingly, due to the elevated GSH levels in cancer cells, treatment with copper-containing oxides selectively reduces GSH levels in malignant cells while largely sparing healthy tissue (Fig. 5a). The reaction site and mechanism of action can be altered by shifting from oxides to copper sulfides (Fig. 4a).^109^ They synthesized hexagonal CuFeS_2_ nanoplates that oxidized GSH *via* a heterogeneous mechanism with competitive *k*_cat_ values, while exhibiting negligible ion release. The authors proposed that the high nucleophilicity of the thiol group in GSH, combined with its strong affinity for Cu (log *K* of Cu(SG) = 26.6 ^110^), enabled the cleaveage of Cu–O bonds in oxides, thereby promoting cation release. This process is likely suppressed in sulfides, where Cu is already coordinated by S^2−^ ligands in a tetrahedral environment.^111^

Another class of nanostructures explored is Prussian Blue Analogues (PBAs) containing copper as an interstitial cation in an N_6_-coordination environment (Fig. 4a).^112^ PBAs are nanoparticles composed of Fe(CN)_6_ units that self-assemble with other metal ions—including Cu—*via* nitrogen coordination.^113^ Bonet-Aleta *et al.*^114^ found that Cu-containing PBAs undergo disassembly in the presence of GSH. However, in this case, copper ions remained coordinated to the Fe(CN)_6_ units during catalysis, accelerating the reaction rate of GSH oxidation (Fig. 5b) compared with previously reported copper nanostructures.^114^ We hypothesized that the high catalytic activity of Cu-PBAs contributed to their observed toxicity against U251-MG cancer cells, while showing no meaningful toxicity toward fibroblasts within the tested concentration range.^114^

In summary, different families of copper-containing nanomedicines have proven effective at catalytically oxidizing GSH under TME conditions.^115^ Nevertheless, the field faces key challenges before advancing to preclinical evaluation. First, the integrity of Cu-containing structures during catalysis is often overlooked, yet recent evidence of their dynamic nature calls for a shift in perspective.

Beyond stability considerations, the ability to release cations should be deliberately addressed during nanostructure design, as an added property that can provide desirable functions to the catalyst. Additionally, it is important to assess whether Cu-containing nanostructures can release ions outside the TME in the presence of other biomolecules besides GSH, potentially causing off-target damage, and how their composition could be tuned to minimize this phenomenon.^109^ Second, Cu cations are inherently bioactive, and cancer cells have evolved precise mechanisms to regulate intracellular Cu. For instance, using activity-based fluorescent probes developed by Chang's group^116^ the response of A549 cells following uptake of Cu-based oxide nanoparticles could be monitored. Specifically, it was observed that cancer cells could lower intracellular Cu(i) levels either *via* GSH-mediated reduction or activation of the NRF2 transcriptional pathway, which regulates the expression of proteins that maintain redox balance.^120^ Notably, the intracellular Cu regulation mechanisms were overwhelmed upon nanoparticle uptake, and cells were unable to effectively manage the released Cu(ii) species, suggesting an exploitable vulnerability. Further studies of this type may reveal new cellular pathways and therapeutic targets associated with catalytic copper.

### 4.2 Oxidation of GSH using nanostructured noble metals

Noble metal catalysts are widely used in both heterogeneous and homogeneous catalysis due to their high turnover frequencies. However, for GSH oxidation, Pt- and Au-based nanoparticles are typically suboptimal, since they form strong interactions with thiol groups leading to surface passivation.^121,122^ Deactivation of noble metals in the biological milieu can be delayed or minimized by catalyst design,^123,124^ but it still represents an important problem for these metals. In contrast, other noble metals like Ir and Ru exhibit greater tolerance toward GSH and retain their catalytic activity, although some evidence suggests partial deactivation particularly with Ir.^125^

Active noble-metal sites have been integrated into nanomedicine formulations as organometallic complexes (Ru),^126,127^ single-atom catalysts (SACs) (Ir^97,128^), or nanoparticles (Ru)^119^ (Fig. 4b). Ru-based organometallic catalysts are known to catalyze thiol oxidation^126,127^ through mechanisms strongly influenced by the ligands coordinated to the metal center.^129^ For instance, Zhang *et al.*^118^ synthesized a Ru–OH complex containing both a phenazoyridine and an iodine ligand, which was nanoencapsulated within a fluoride-rich polymer to promote O_2_ delivery to hypoxic TME regions (Fig. 5c). The phenazoyridine moiety facilitated electron transfer and initiated a reaction with GSH *via* thiol addition to the azo bond, while the iodine ligand helped to reduce undesired ligand exchange with the medium. The encapsulated O_2_ acted as a fuel for GSH oxidation, significantly enhancing the catalyst's *in vivo* efficacy (Fig. 5c).

A similar Ir-based complex,^130^ also bearing phenazoyridine and iodine ligands, catalyzed GSH oxidation without the need for nanoencapsulation. The catalytic mechanism again involved thiol attack on the azo bond, forming GSSG. However, at high GSH concentrations (10 mM), the integrity of the Ir complex was compromised by the formation of bimetallic Ir–GSH adducts, leading to catalyst deactivation. Cheng *et al.*^97^ synthesized Ir–N_4_ SACs by assembling a nanoscale metal–organic framework (MOF) containing Ir atoms, followed by pyrolysis to generate well-defined Ir–N_4_ sites. These catalysts oxidized GSH without deactivation in cellular environments. Furthermore, functionalization with red blood cell membranes enhanced tumor targeting and improved biodistribution. In the field of Ru-based nanomedicine, the prevailing strategy has involved the direct encapsulation of active Ru complexes within nanoparticles.^131,132^ The only reported example of a Ru-based nanomaterial involves RuO_2_ nanoparticles doped with Pt(iv) complexes displaying multiple enzyme-like activities,^119^ including GSH oxidase and catalase-like activities. These nanoparticles successfully reduced GSH even under hypoxic conditions through synergistic catalysis.

In summary, noble metal-based nanocatalysts are emerging as effective tools for GSH oxidation; however, their development is still limited, and concerns remain about systemic toxicity, especially in theranostic applications. Also, their application in biological environments including the TME will in most cases require improving their resistance to deactivation by biomolecule poisoning.^124,133^ A central question also remains as to whether the catalytic benefits of noble metals outweigh those of more traditional transition metals such as copper, an issue that must be addressed on a case-specific basis. In this context, standardized *in vitro* catalytic assays and the development of additional physicochemical probes will be essential to unravel intracellular mechanisms of action and guide future research priorities.

### 4.3 Catalytic oxidation of GSH using light or ultrasound as external stimuli

The major advantage of using an exogenous signal lies in the temporal (and sometimes spatial) control it provides. The clinical gains would be obvious if the therapeutic action can be restricted to the tumor region (or at least to a certain time during which the concentration of the therapeutic agent is still minimal in healthy tissues); a more precise activation of nanomedicines within cancerous tissue would be obtained, while minimizing undesired secondary effects. Most nanomedicine-based strategies leveraging this concept rely on photothermal (PT) nanoparticles that locally raise the temperature in tumors upon NIR laser irradiation, leveraging the highly directional nature of laser light to restrict impact in other areas.^134^ However, beyond thermal effects chemical reactions can also be catalytically triggered using semiconductor nanoparticles with well-defined band structures (Fig. 4c). Depending on the nature of applied stimulus, the process is referred to as photocatalysis (when using light) or sonocatalysis (when using ultrasound). Semiconductor nanoparticles possess an electronic structure characterized by two distinct bands (Fig. 4c): the valence band—the highest energy band fully occupied by electrons at absolute zero—and the conduction band, which lies above the valence band and is typically empty or partially filled in insulators and semiconductors at low temperatures. These bands are separated by an energy barrier known as the band gap (*E*_g_). When an inorganic semiconductor is exposed to light with energy equivalent to its *E*_g_, electrons in the valence band are excited to the conduction band, generating an electron–hole pair (h^+^/e^−^) in the process. The nucleophilic thiol group of GSH can then react with the photogenerated holes (h^+^), undergoing catalytic oxidation and enabling continuous, localized depletion of GSH in tumor tissues. This approach provides an additional degree of selectivity through the spatiotemporal control of laser irradiation. To demonstrate this principle, Zhao *et al.*^135^ designed a semiconductor nanostructure composed of WO_2.41_@SnS_1.68_ with a band gap suitable for near-infrared (NIR) light (808 nm) excitation—a wavelength particularly favourable in biomedicine due to its comparatively deeper tissue penetration.^137^ Upon NIR laser activation, the photocatalyst depleted 40 µmol of GSH per gram of catalyst, leading to a significant antitumor effect (Fig. 5d).

Ultrasound (US) offers an alternative energy source with even greater tissue penetration depth (<10 cm),^138^ making it particularly attractive for treating deep-seated tumors such as pancreatic cancer.^139^ US waves generate nanoscale cavitation bubbles that collapse to create localized extreme conditions, with transient temperatures reaching up to 5000 K and pressures exceeding 1000 atm. These conditions can promote electron excitation across the band gap, analogous to photoabsorption in photocatalysis (Fig. 4c). Most reported sonocatalysts generate ROS under US stimulation,^140^ but only a few have demonstrated activity against GSH.^141^ A notable example is composed of KBiO_3_ nanosheets with a tuned *E*_g_ of 1.9 eV, synthesized by Peng *et al.*,^142^ which exhibited ultrasound-catalytic activity toward GSH depletion in cells. Nevertheless, the underlying mechanism remains insufficiently understood and requires further investigation to enable precise tuning of future nanomedicines.

### 4.4 Copper nanostructures catalyze the transamination of GSH and GSSG

In addition to the previously discussed role of Cu^2+^ as a homogeneous catalyst for GSH oxidation, we have recently demonstrated the active role of Cu-containing nanoparticles to deplete GSH levels beyond simple oxidation. Specifically, we found that CuFe_2_O_4_ nanoparticles acted as copper reservoirs, releasing Cu^2+^ ions that catalyze the transamination of both GSH and GSSG (Fig. 5e).^136^ This reaction requires an α-keto acid and an amino acid, with Cu^2+^ catalyzing the exchange of their respective oxo and amino groups. Intracellularly, this process may occur using pyruvate as the α-keto acid and any available amino acid as the substrate. Importantly, GSH and GSSG are γ-peptides, meaning at least one of their peptide bonds is formed *via* a functional group on the side chain, leaving a free α-amino acid moiety susceptible to Cu-catalyzed transamination (Fig. 5e).

This uncommon catalytic reaction offers two major therapeutic advantages: (i) it does not require O_2_, which is often scarce in the hypoxic regions of the TME, and (ii) it generates a product—α-keto GSSG—that cells cannot readily reuse, unlike GSSG, which can be reconverted to GSH by the enzyme glutathione reductase (Fig. 5e).

Looking ahead, we envision the transamination reaction as a promising tool to deplete GSH/GSSG in cancer cells under challenging physiological conditions. Therefore, significant efforts should be directed toward integrating organic chemistry, chemical biology, and nanomedicine to discover new GSH reactivities beyond oxidation that can be exploited by nanocatalysts for cancer therapy. Catalytic nanomedicines can cyclically oxidize GSH, a phenomenon widely shown to be particularly harmful to cancer cells. However, the implementation of this class of nanomedicines faces multiple significant challenges. First, most of these systems are composed of oxidized transition metals, which can entail potential off-target toxicity. Numerous studies have shown that these materials can generate active, catalytic ions under conditions such as the acidic environment of the TME. Yet this release could also occur inside lysosomes, especially considering that many nanomaterials are internalized *via* the lysosomal route,^106,143^ ultimately leading to potential undesired off-target toxicity. Beyond pH, other biomolecules can also affect the integrity of transition-metal-based nanoparticles, generating dissolved cations through leaching. This is the case for copper and GSH, as we show in Section 3.2, but also for other substances—such as most amino acids^136,144^—that could similarly cause off-target damage in healthy tissues. Therefore, it remains critical to test the toxicity, metal release and/or catalytic activity of the tested nanomedicines also in healthy cell models such as fibroblasts or under general biological conditions (*e.g.* serum, culture medium). Selectivity also remains a second-order concern, as catalytic nanomedicines should ideally target the overexpressed GSH in tumours to maximize therapeutic specificity. Nevertheless, recent reports explore the oxidation of other intracellular thiols, such as cysteine.^145^ We predict that tuning nanomedicine chemistry to be selective for GSH over other thiols will be extremely challenging, and that efforts may be more effective when focused on improving cancer-cell targeting. Since GSH is orders of magnitude more concentrated in the cytosol than other thiols,^146^ it will ultimately serve as the preferred substrate for the nanocatalyst.

## 5 Conclusions and outlook

The strong contribution of GSH to tumor chemoresistance, combined with its multifunctional biological roles, makes it an attractive target for the development of next-generation nanomedicines. The high demand for GSH in cancer cells to sustain rapid proliferation can be exploited by nanotherapeutics that react with GSH either stoichiometrically or catalytically as part of a multifunctional treatment strategy. A new generation of nanoparticles now incorporates GSH-responsive bonds, enabling locally triggered release of therapeutics, thereby reducing premature biodegradation en route to the tumor. Over the past decade, a wide variety of nanomedicines leveraging GSH overexpression in the TME have been developed and evaluated in preclinical cancer models. However, the rapid expansion of materials and strategies has made it increasingly challenging to draw clear insights or to identify the most effective nanostructures, combinations or mechanisms of action.

Both stoichiometric and catalytic approaches have shown to effectively deplete intracellular GSH in cancer cells. At present, the design of stoichiometric nanomedicines allows, to a greater extent, their combination with multiple drugs, owing to the spatiotemporal control of drug release enabled by GSH-responsive reactions. This strategy is particularly well developed in the case of GSH-sensitive nanostructures (Fig. 2b), for which many examples have been reported. In contrast, the catalytic approach, while still less developed, has the potential to achieve stronger GSH depletion due to its cyclic mechanism. In principle, even a small amount of nanocatalyst reaching the tumor can repeatedly deplete GSH until the substrate is exhausted or the catalyst becomes deactivated. However, the lack of available kinetic data on GSH depletion prevents a clear verdict regarding which approach or material could be more effective in therapy, and at this stage both strategies face very similar challenges for future clinical translation. We emphasize the urgent need to establish standardized and well-characterized reaction kinetics with GSH for each type of nanostructure, regardless of whether the process is catalytic or stoichiometric. Such standardization would generate fundamental data to reliably ascertain which nanostructures most efficiently deplete GSH—and the mechanistic basis for their effectiveness. At the cellular level, there is strong consensus and experimental evidence to support that cancer cells are more sensitive to GSH depletion. Nonetheless, it remains essential to define the degree of sensitivity across different cancer cell types and investigate the underlying causes, as this may reveal new vulnerabilities related to GSH dysregulation.

Advancing GSH-targeting nanomedicines toward clinical translation will require the integration of cross-disciplinary knowledge spanning materials science, chemical biology, and catalysis. Such knowledge will provide the blueprint for designing the next generation of GSH-targeting nanostructures capable of selectively exploiting redox dysregulation in cancer.

Despite clear benefits observed in preclinical studies, the clinical translation of GSH-targeting nanomedicines is still challenging. Some fundamental questions remain unanswered, such as: What is the optimal approach in terms of GSH-depleting kinetics? What are the average pharmacokinetic values, and how can they be changed with different nanomedicine designs? Is there a specific type of cancer where these nanomedicines are particularly effective? The situation is reminiscent of the PROTACs (Proteolysis Targeting Chimeras) field, where almost 1100 candidates with reported cellular activity data have been described, and around 50 are in clinical trials—including 2 in Phase III. Yet, the overall success rate remains modest, even with excellent activity profiles and a deep understanding of their chemistry, mechanism of action, and pharmacokinetics. Similarly, most GSH-depleting nanomedicines studied to date have focused on evaluating isolated go/no-go activities rather than building a broader, comparative framework that connects different nanomedicines and treatment strategies. This has led to a limited vertical progress in the field.

**Fig. 6:**
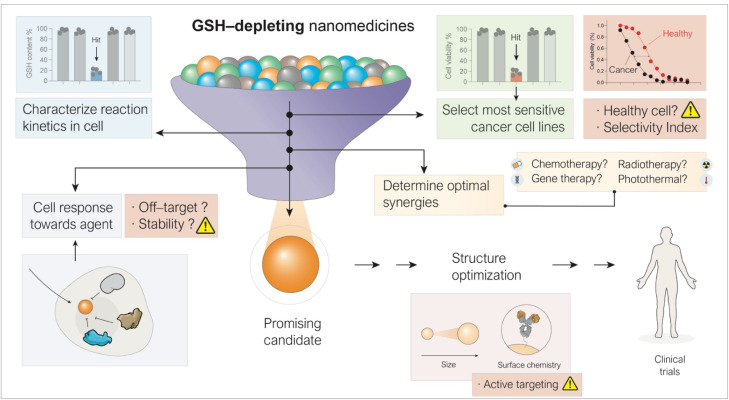
Potential considerations to strengthen the evaluation of promising candidates include: (i) quantifying the GSH depletion rate in cells for all candidates, (ii) screening across cell line libraries to identify the most sensitive cancer types, (iii) characterizing the target cell's response to the nanomedicine, and (iv) determining optimal synergies with co-therapeutic agents.

Moving forward, we highlight five key aspects which should be explored in depth to significantly accelerate progress toward clinical translation (Fig. 6).

### 5.1 Characterization of kinetics and mechanisms in cells

Deeper characterization of reaction rates in cells and mechanisms leading to GSH depletion is required to establish the most optimal modality (either stoichiometric or catalytic) and design of the GSH depletion strategy. Accurate kinetics are essential, since rates must be sufficiently high to overcome the natural cellular mechanisms to replenish GSH levels. Current cellular assays - typically based on colorimetric kits- are suitable for semi-quantitative changes in the intracellular GSH pool following treatment but insufficient to ascertain true kinetics. We recommend extraction of remaining intracellular GSH and quantification by HPLC-MS coupled with measurements of nanomedicine uptake over time, to derive efficacy parameters that allow direct comparison across platforms.^106^

### 5.2 Consider cellular response to GSH depletion and off-target metal release

It would be of particular interest to evaluate how cells counteract GSH depletion, as detoxification pathways may attenuate the effectiveness of highly active catalysts. This was observed in one of our recent studies, conducted in collaboration with Prof. Chris Chang's group, involving Cu(ii) cations released from Cu_2_FeO_4_ nanoparticles in A549 cells.^120^ We studied this cell response by using activity-based fluorescent probes which can selectively bind and sense copper cations.^147,148^ After nanoparticle internalization and copper release, the cells activated specific mechanisms to regulate the Cu(i) pool, thereby reducing the amount of available catalyst for GSH oxidation. This experimental observation led us to conclude that a catalyst which appears less active in test tube assays where there are no cells present, but goes unnoticed by cells under intracellular conditions, may ultimately result in greater GSH depletion.

There are multiple fluorescent probes that enable the monitoring and tracking of metal ions in living systems and could potentially be used to study target and off-target metal release, including probes for Fe(iii), Fe(ii), Cu(ii), Cu(i), Mn(ii), and Co(ii), among others. A comprehensive list of such biosensors was compiled by Grover *et al.* in a recently published review.^116^ This opens up a toolbox available to researchers working with nanomedicines based on these metals to make use of these tools to study both cellular responses to the metals and their potential off-target release in cell culture and in mice.

### 5.3 Identification of sensitive cancer cell lines

Not all tumors display the same basal GSH levels or sensitivity to GSH depletion. Systematic screening of different nanomedicine modalities across libraries of cancer cell lines will be essential to identify the most responsive cancer types. In addition, as the library of potential anti-cancer agents expands, identifying candidates with genuine translational potential becomes increasingly challenging. In this context, the Selectivity Index (SI)—defined as the ratio of the IC_50_ value in non-cancerous cells to that in cancer cells—serves as a critical quantitative metric for evaluating the safety window of new formulations, yet it is not broadly reported in most published works in this field. A high SI (typically >2–3) serves as a robust initial filter. However, special caution is necessary when using this parameter to draw conclusions in the field of nanomedicine. Unlike small-molecule chemotherapeutics, which rely primarily on intrinsic molecular selectivity, the safety profile of nanomedicines is strongly influenced by biological processes that take place only *in vivo*—such as the enhanced permeability and retention effect, extravasation from blood vessels or immune system response, including removal of nanoparticles by macrophages in the liver and spleen—which are absent or minimized in standard 2D culture assays. Consequently, a modest *in vitro* SI does not necessarily preclude clinical utility if the carrier incorporates a sophisticated targeting mechanism (*e.g.* peptide-based targeting or extracellular vesicle enhanced delivery) or specific activation mechanisms (*e.g.* local pH or response to near-infrared light). Thus, actively targeted or pH-responsive systems may exhibit indiscriminate cytotoxicity in conventional monolayer assays, yet demonstrate a superior therapeutic index *in vivo* due to preferential accumulation or activation in the tumor microenvironment.^149,150^ Therefore, while reporting SI data is important for transparency and benchmarking, in the context of nanomedicine it should be evaluated not in isolation, but in conjunction with the specific targeting strategy and delivery mechanism employed, as highlighted in previous Perspective articles.^151^ In this context, modern high-throughput screening techniques will play a critical role in generating large datasets for this purpose.

### 5.4 Mapping therapeutic synergies

GSH depletion has been combined with photothermal therapy, radiotherapy, chemotherapy or immunotherapy among others. Yet it remains unclear which combinations offer the strongest benefit. Rigorous evaluation of co-treatments, again supported by modern high-throughput screening techniques, will play a critical role in identifying the most promising synergies and designing rational therapeutic protocols.

### 5.5 Novel nanoformulations and active targeting

Addressing these priorities will provide the foundation for a more systematic and quantitative framework to compare different nanomedicine platforms. The resulting data will also guide the optimization of structural parameters—such as particle size, surface chemistry, pharmacokinetics, smart delivery, biomimicking, and stealth coatings—that strongly influence therapeutic performance. Ideally, when using metal-based nanomedicines to deplete GSH, the off-target release of ions in healthy tissues should be avoided. However, developing mechanisms that prevent metal release in healthy tissue is extremely challenging, even when deploying surface functionalization, polymer coatings, or other physical barriers. The best solution for this class of nanomedicines seems the combination with an effective active targeting strategy to maximize tumor accumulation. In this way, if not completely prevented, at least the proportion of released cations reaching off-target tissues will be strongly minimized. In this regard, active recognition of targets by moieties such as antibodies or peptides attached to nanoparticles is compromised by the protein corona formed around nanoparticles in biological environments.^152^ In addition, these attached molecules may actually enhance recognition and uptake by macrophages.^153,154^ Because of this, other avenues are being explored, including camouflaging the nanoparticles with biological membranes, red blood cells, platelets, immune cells, tumor cells, and viruses,^155^ or enclosing them within extracellular vesicles,^156,157^ which present better potential to evade macrophage capture while improving targeting selectivity. Ultimately, advancing GSH-targeting nanomedicines toward clinical translation will require close integration of materials science, chemical biology, and catalysis. Such cross-disciplinary efforts will enable the rational design of the next generation of nanomedicines capable of selectively exploiting redox dysregulation in cancer, paving the way for transformative clinical applications.

## Author contributions

J. B.-A.: conceptualization, writing – original manuscript, writing – review and editing. A. M.: writing – review and editing. J. L. H.: writing – review and editing, analysis and interpretation. J. S.: writing – review and editing, analysis and interpretation.

## Conflicts of interest

The authors declare that there are no conflicts to declare.

## Data Availability

No primary research results, software or code have been included and no new data were generated or analysed as part of this review.
